# The cumulative effect of the combined action of miR-101 and curcumin in a liposome on a model of Alzheimer’s disease in mononuclear cells

**DOI:** 10.3389/fncel.2023.1169980

**Published:** 2023-04-21

**Authors:** Victoria Vasilevna Sokolik, Olga Grigorievna Berchenko

**Affiliations:** Department of Neurophysiology, Immunology and Biochemistry, State Institution “Institute of Neurology, Psychiatry and Narcology of the National Academy of Medical Sciences of Ukraine”, Kharkiv, Ukraine

**Keywords:** Alzheimer’s disease, miR-101, curcumin, liposomes, mononuclear cells, cytokines

## Abstract

The leading pathological mechanisms of Alzheimer’s disease (AD) are amyloidosis and chronic inflammation. The study of new therapeutic drugs of the corresponding action, in particular miRNAs and curcominoids, as well as methods for their packaging, is topical. The aim of the work was to study the effect of miR-101 + curcumin in a single liposome in a cellular AD model. AD model was made by incubating a suspension of mononuclear cells with aggregates of beta-amyloid peptide 1–40 (Aβ40) for 1 h. The effect of the subsequent application of liposomal (L) preparations miR-101, curcumin (CUR), and miR-101 + CUR was analyzed over time of 1, 3, 6, and 12 h. A decrease in the level of endogenous Aβ42 under the influence of L(miR-101 + CUR) was revealed during the entire incubation period (1–12 h), the first part of which was overlapped due to inhibition of mRNA^APP^ translation by miR-101 (1–3 h), and the second-by inhibition of mRNA^APP^ transcription by curcumin (3–12 h), the minimum concentration of Aβ42 was recorded at 6 h. The cumulative effect of the combination drug L(miR-101 + CUR) was manifested in the suppression of the increase in the concentration of TNFα and IL-10 and a decrease in the concentration of IL-6 during the entire incubation period (1–12 h). Thus, miR-101 + CUR in one liposome enhanced each other’s antiamyloidogenic and anti- inflammatory effects in a cellular AD model.

## Introduction

The loss of neural connections in the Alzheimer’s disease (AD) brain is mainly due to the disruption of signaling pathways that affect both synaptic plasticity and dendritic function, two critical regulators of cognitive processes (Abuelezz et al., 2021). At the molecular level, studies show that beta-amyloid peptide (Aβ) and Tau (τ) pathologies cause progressive axonal degeneration and severe disruption of downstream synaptic processes (Pereira et al., 2021). Moreover, Aβ and τ aggregates cause an increase in the immune response of microglia, as well as a violation of the functionality of astrocytes, which ultimately contributes to cognitive decline in AD (Fakhoury, 2018).

Current treatments for AD currently focus on the use of acetylcholinesterase inhibitors designed to inhibit the enzyme acetylcholinesterase to increase acetylcholine levels (Mehta et al., 2012). However, these drugs can only relieve AD symptoms and have harmful side effects that limit success (Iqbal et al., 2010). Therefore, there is an urgent need for an effective therapeutic agent for the treatment of AD.

About 70% of experimentally detected miRNAs are expressed in the brain, where they regulate neurite outgrowth, dendritic spine morphology, and synaptic plasticity. A growing body of research shows that miRNAs are deeply involved in synaptic function and specific signals during memory formation (Reddy et al., 2017). This was a key point in considering the critical miRNAs that are being studied in AD. MicroRNA dysfunctions are increasingly recognized as a major factor influencing AD through deregulation of genes involved in the pathogenesis of AD. MiRNAs that directly target the APP support a role for miRNAs in the pathogenesis of AD. Reduced levels of miR-101 in the AD brain are reported, consistent with *in vitro* studies in which miR-101 inhibition increased APP levels (Siedlecki-Wullich et al., 2021). And vice versa: exogenous miR-101 caused inhibition of mRNA^APP^ translation, reducing the level of endogenous Aβ42 and the inflammatory cytokine response (Sokolik et al., 2021).

Recent reports also suggest a therapeutic potential for CUR in the pathophysiology of Alzheimer’s disease (Hamaguchi et al., 2010). CUR has been reported in *in vitro* studies to inhibit Aβ aggregation and Aβ-induced inflammation, as well as β-secretase and acetylcholinesterase activity (Ono et al., 2004; Ahmed and Gilani, 2009; Sokolik and Shulga, 2016). In *in vivo* studies, oral or nasal administration of CUR resulted in inhibition of Aβ deposition, Aβ oligomerization, and tau phosphorylation in the brain of AD animal models, as well as improvement in behavioral disturbances and cognitive characteristics (Garcia-Alloza et al., 2007; Sokolik and Shulga, 2015). These data suggest that CUR may be one of the most promising compounds for the development of AD treatments.

Another problem of AD therapy is the difficulty of effective and non-invasive drug delivery directly to the brain. However, growing advances in nanotechnology (Mageed et al., 2021) and targeted delivery of biological materials have been successfully reported in some animal AD models.

In our previous studies, the predominantly anti-amyloidogenic and/or anti- inflammatory effects of liposomal preparations of CUR and miR-101 have been established individually in animal and cellular models of Alzheimer’s disease (Sokolik and Shulga, 2015, 2016; Sokolik et al., 2017, 2019, 2021). Therefore, it is logical that the aim of this study was to study the effect of miR-101 + CUR in a single liposome in an experimental AD model *in vitro*.

## Materials and methods

A cellular Alzheimer’s disease (AD) model was made *in vitro* on a suspension of human mononuclear cells (*n* = 3 samples). The ethics committee of the SI “Institute of Neurology, Psychiatry and Narcology of the National Academy of Medical Sciences of Ukraine,” Kharkiv, Ukraine approved this study (Protocol no. 12-à, 12.12.2019). Written informed consent from volunteer donors was obtained at the time of blood sampling. Mononuclear cells were isolated using a Ficoll-Urografin density gradient, washed three times with 0.9% NaCl, and resuspended in RPMI medium to a concentration of 12,000 × 10^9^ cells per liter.

Beta Amyloid Peptide (1–40) (Human), Abcam, Cambridge, UK was dissolved in double- distillate water to the concentration of 1.5 mmol/L and aggregated at 37°C during 24 h. Large conglomerates of Aβ40 were dispersed by ultrasound and sterilized intermediary before adding to the suspension of mononuclear cells. The suspension of Aβ40 aggregates was added to the incubation medium RPMI with mononuclear cells in a 1:17 dose.

The liposomal forms of miR-101 (miR-101-3p, SPF Sintol LLC, and RF), CUR (Sigma-Aldrich, St. Louis, MO, USA) and miR-101 + CUR was obtained using the lipid injection method. The diameter of liposomes (composed of phospholipid/cholesterol) was calibrated in an extruder using additional membranes with 100 nm openings. The concentration of microRNA in the suspension of liposomes was 12.5⋅10^18^ molecules per liter, the CUR concentration was 0.7 g per liter. At the same time, liposomal preparations were added to the incubation medium at a ratio of 1:50. Nucleotide sequence of miR-101: UACAGUACUGUGAUAACUGAA.

Four experimental groups were created for each from three different samples of mononuclear suspensions:

Control: mononuclear cells + 0.9% NaCl (1 h) + 0.9% NaCl Group L(miR-101): mononuclear cells + Aβ40 (1 h) + L(miR-101).

Group L(miR-101 + CUR): mononuclear cells + Aβ40 (1 h) + L(miR-101 + CUR) Group L(CUR): mononuclear cells + Aβ40 (1 h) + L(CUR), Aβ40 aggregates (except for Control) were added to these cells, and after 1 h of incubation at 37°C, liposome suspensions miR-101, miR-101 + CUR, and CUR were added, respectively. Aliquots (*V* = 300 μl) for the determination of the concentration of Aβ42 and cytokines were taken at the beginning of the experiment, after 1 h of incubation with Aβ40 and at the dynamics of the time after adding the suspension of liposomal reagents: on the 1st, 3rd, 6th, and 12th h. The control was given the corresponding volume of saline. Samples were negatively frozen and stored until ELISA simmered at −80°C.

Enzyme-linked immunosorbent assay for Aβ42 and cytokines (TNFα, IL-6, IL-10) concentration was performed according kit manuals for the Human Aβ 1–42 (Amyloid Beta 1–42) ELISA Kit Elabscience Bio-technology Inc., USA, α-TNF-EIA-BEST, Interleukin-6-EIA- BEST and Interleukin-10-EIA-BEST, RF. The absorbance of samples was read with a GBG Stat FAX 2,100 microplate analyzer (USA) at 450 nm with wavelength correction at 630 nm. The EIA data were represented in picograms per mL (pg/mL). To identify the specificity of the effect of Aβ40 and liposomal preparations miR-101, miR-101 + CUR, and CUR, the data of the L(miR-101), L(miR-101 + CUR), and L(CUR) groups were normalized to the corresponding indicators of the control group in dynamics research and are expressed in%.

Data are presented as mean ± standard deviation (SD). For comparison of non-parametric data sets the Mann-Whitney *U*-test. The results were considered statistically significant at *P* < 0.05.

## Results

**FIGURE 1:**
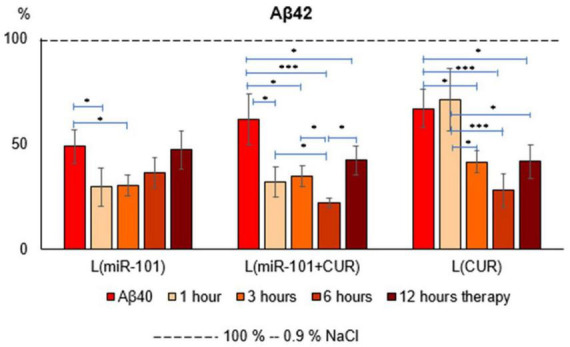
Diagrams of the relative content of Aβ42 in suspension of mononuclear cells after 1 h of incubation with aggregates of Aβ40 and subsequent incubation over time for 1, 3, 6 and 12 h with liposomal forms of miR-101, CUR, and miR-101 + CUR. The vertical axis is the concentration of Aβ42 in the target samples normalized to the corresponding concentration of this substance in the suspension of mononuclear cells incubated with saline, %. *P < 0.05, ***P < 0.001.

The liposomal form of miR-101 + CUR revealed the most effective (from the first hour of incubation by an average of 67% over the entire period) and long-term (within 12 h) suppression of the Aβ42 level in mononuclear suspension, caused by the preliminary administration of Aβ40 aggregates (Figure 1) according to compared with the action of miR-101 and CUR in liposomes individually. Thus, miR-101 in liposomes caused a decrease in the level of Aβ42 only during the first 3 h of incubation and, on average, by 70% during this time, while the effect of liposomal CUR actualized during 3–12 h of incubation, i.e., was more delayed.

The greatest effect of miR-101 + CUR liposomal preparation turned out to be at 6 h of incubation of mononuclear cells with the AD model (Figure 1). It was most compelling when compared to liposomal miR-101 or CUR individually in the same term. These data characterize the primary antiamyloidogenic action of miRNAs and only an indirect and delayed effect of CUR.

**FIGURE 2:**
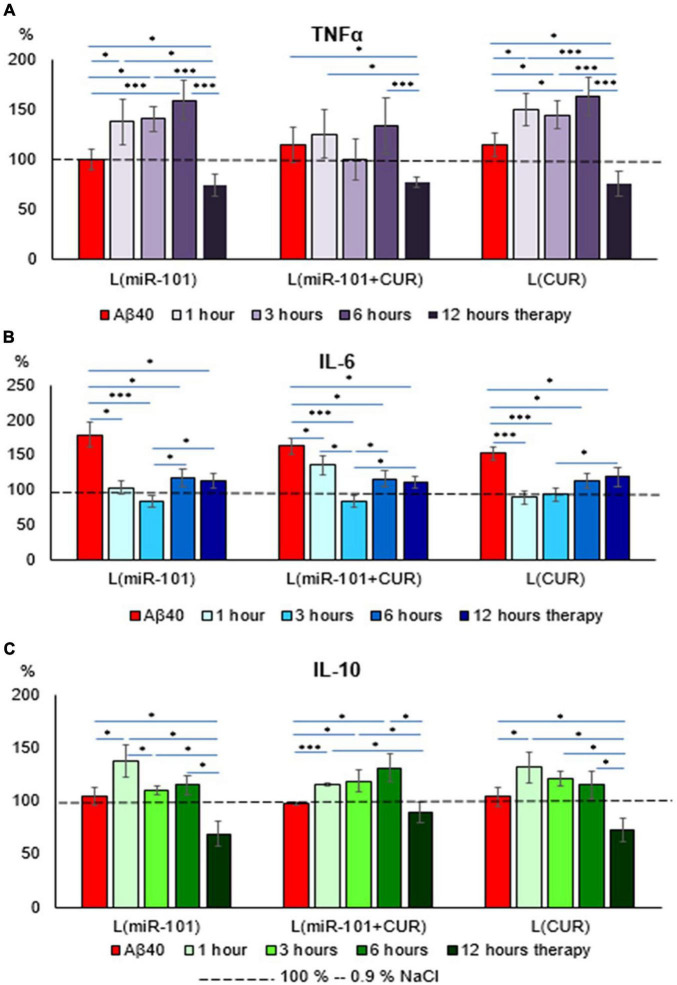
Diagrams of the relative content of cytokines: TNFα **(A)**, IL-6 **(B)**, and IL-10 **(C)** in mononuclear cell suspension after 1 h of incubation with Aβ40 aggregates and subsequent incubation over time 1, 3, 6, and 12 h with liposomal forms of miR-101, CUR, and miR-101 + CUR. The vertical axis is the concentration of the corresponding cytokine in the target samples normalized to the corresponding concentration of this substance in the suspension of mononuclear cells incubated with saline, %. *P < 0.05, ***P < 0.001.

Liposomal preparations of miR-101 or CUR individually during the first 6 h of incubation failed to prevent an increase in TNFα concentration in the suspension of mononuclear cells activated by Aβ40 aggregates, in contrast to liposomes with miR-101 + CUR (Figure 2A). A decrease in the concentration of this cytokine by 12 h of incubation cannot be considered a specific anti-inflammatory effect of liposomes with microRNA and CUR, since TNFα is a cytokine of the first wave of the inflammatory cascade and its level decreases physiologically by this time, as well as IL-10 (Figure 2C). What cannot be said about the concentration dynamics of the ambivalent representative of the second wave of the cytokine cascade-IL-6 (Figure 2B). It was shown that all liposomal forms of reagents specifically reduced the concentration of IL-6 during the entire incubation period (1–12 h), which illustrates their anti-inflammatory effect. The dynamics of IL-10 concentration presented in Figure 2C shows that miR-101 + CUR in liposomes provides a stable increase in the level of this anti- inflammatory cytokine in the range of 1–6 h of incubation, while only miR-101 or only CUR in liposomes is effective only for the first hour of incubation.

## Discussion

Alzheimer’s disease is a multifactorial metabolic disorder of the central nervous system, so a single drug with a narrowly focused symptom-corrective action cannot be a panacea for it. From a large pool of miRNAs involved in the pathogenesis of AD (more than 40, 16 of which have diagnostic potential in humans), miR-101 was selected.

Deficiency of the latter leads to uncontrolled induction of APP synthesis, the excess of which is converted by beta-site APP cleaving enzyme 1 (BASE1) and γ- secretase into aggregate active Aβ (Vilardo et al., 2010). Altered expression of miR-101 was found in patients with AD. Two independent miRNA expression profiles (Hébert et al., 2008; Nunez-Iglesias et al., 2010) showed that miR-101 is down-regulated in the human cortex in AD, indicating that miR-101 down-regulation may play a role in the development of AD. This hypothesis is consistent with our previous results, which indicate a direct role for miR-101 in the control of APP translation and Aβ accumulation (Sokolik et al., 2021).

Liposomal miR-101 in mononuclear cell suspension caused a decrease in Aβ42 levels only for the first 3 h of incubation, while together with CUR, the effect lasted 12 h (Figure 1), since CUR came into play just starting from 3 h of incubation. This dynamic is since miR-101 has a direct inhibitory effect on the translation of mRNA^APP^, while CUR affects indirectly: by inhibiting the inflammatory cytokine cascade. These data are consistent with previously published results that FAM-labeled miR-101 in liposomes is actively absorbed by mononuclear cells during the first 1–2 h of incubation, and then the intensity of the fluorescent signal decreases by half by 3 h of incubation (Sokolik et al., 2021).

Most likely, degradation of miR-101 occurs. Our other study revealed that CUR causes a decrease in mRNA^APP^ expression precisely by 3 h of incubation (Sokolik and Shulga, 2016) and a corresponding decrease in Aβ concentration. Thus, miR-101 has a rapid and direct effect on mRNA^APP^ translation, while CUR has a slow and indirect effect on its transcription. Therefore, their combined use in an experimental AD model had the most effective antiamyloidogenic effect.

Aβ is a representative of the ancient peptide defense system of the brain-antimicrobial peptides (AMPs), which can react faster and first of the cellular immunity systems and the cytokine cascade, activating the latter (Kumar et al., 2016; Moir et al., 2018; Pastore et al., 2020). In the present study, miR-101 in liposomes showed an indirect effect on the dynamics of the cytokine response (except for IL-6), which is most likely due to an inhibitory effect on the level of endogenous Aβ. CUR, in turn, is considered as a direct negative regulator of IL-6 inflammatory signaling pathways due to suppression of NF-kappaB activation (Ghandadi and Sahebkar, 2017). Therefore, of greatest interest is the revealed cumulative effect of their combined action miR-101 + CUR in one liposome on an experimental AD model. Namely: suppression of the increase in the concentration of TNFα and IL-10 and a decrease in the concentration of IL-6 during the entire incubation period (1–12 h). Undoubtedly, the subtle regulatory mechanisms of this effect require further detailed study, but the obtained fact inspires optimism regarding the advisability of using the liposomal form of miR-101 with CUR for the treatment of Thus, miR-101 + CUR in one liposome enhanced each other’s antiamyloidogenic and anti-inflammatory effects in a cellular AD model.

## Data availability statement

The original contributions presented in this study are included in the article/supplementary material, further inquiries can be directed to the corresponding author.

## Ethics statement

The studies involving human participants were reviewed and approved by the Ethics Committee of the SI “Institute of Neurology, Psychiatry and Narcology of the National Academy of Medical Sciences of Ukraine”, Kharkiv, Ukraine (Protocol No. 12-à, 12.12.2019). The patients/participants provided their written informed consent to participate in this study.

## Author contributions

VS carried out the entire experimental part of the work, statistical processing of the results, formulated the concept and task of the study, and prepared the manuscript for publication. OB took an active part in the discussion of the results and the formulation of the conclusions of the manuscript. Both authors contributed to the article and approved the submitted version.

## References

[B1] Abuelezz N. Z. Nasr F. E. AbdulKader M. A. Bassiouny A. R. Zaky A. (2021). MicroRNAs as Potential Orchestrators of Alzheimer’s Disease-Related Pathologies: Insights on Current Status and Future Possibilities. *Front. Aging Neurosci.* 13:743573. 10.3389/fnagi.2021.743573 34712129 PMC8546247

[B2] Ahmed T. Gilani A. H. (2009). Inhibitory effect of curcuminoids on acetylcholinesterase activity and attenuation of scopolamine-induced amnesia may explain medicinal use of turmeric in Alzheimer’s disease. *Pharmacol. Biochem. Behav.* 91 554–559. 10.1016/j.pbb.2008.09.010 18930076

[B3] Fakhoury M. (2018). Microglia and Astrocytes in Alzheimer’s Disease: Implications for Therapy. *Curr. Neuropharmacol*. 16 508–518. 10.2174/1570159X15666170720095240 28730967 PMC5997862

[B4] Garcia-Alloza M. Borrelli L. A. Rozkalne A. Hyman B. T. Bacskai B. J. (2007). Curcumin labels amyloid pathology in vivo, disrupts existing plaques, and partially restores distorted neurites in an Alzheimer mouse model. *J. Neurochem.* 102 1095–1104. 10.1111/j.1471-4159.2007.04613.x 17472706

[B5] Ghandadi M. Sahebkar A. (2017). Curcumin: An Effective Inhibitor of Interleukin-6. *Curr. Pharm. Des*. 23 921–931. 10.2174/1381612822666161006151605 27719643

[B6] Hamaguchi T. Ono K. Yamada M. (2010). Curcumin and Alzheimer’s Disease. *CNS Neurosci. Therap.* 16 285–297. 20406252 10.1111/j.1755-5949.2010.00147.x PMC6493893

[B7] Hébert S. S. Horré K. Nicolaï L. Papadopoulou A. S. Mandemakers W. Silahtaroglu A. N. (2008). Loss of microRNA cluster miR-29a/b-1 in sporadic Alzheimer’s disease correlates with increased BACE1/β-secretase expression. *Proc. Natl. Acad. Sci. U.S.A.* 105 6415–6420. 10.1073/pnas.071026310 18434550 PMC2359789

[B8] Iqbal K. Liu F. Gong C.-X. Grundke-Iqbal I. (2010). Tau in Alzheimer disease and related tauopathies. *Curr. Alzheimer Res*. 7 656–664. 10.2174/156720510793611592 20678074 PMC3090074

[B9] Kumar D. K. Choi S. H. Washicosky K. J. Eimer W. A. Tucker S. Ghofrani J. (2016). Amyloid-β peptide protects against microbial infection in mouse and worm models of Alzheimer’s disease. *Sci. Transl. Med*. 8:340ra72. 10.1126/scitranslmed.aaf1059 27225182 PMC5505565

[B10] Mageed A. H. Emad A. Mohamed S. A. Abuelezz N. (2021). The tiny big world of solid lipid nanoparticles and nanostructured lipid carriers: An updated review. *J. Microencapsul.* 39 1–42. 10.1080/02652048.2021.2021307 34958628

[B11] Mehta M. Adem A. Sabbagh M. (2012). New acetylcholinesterase inhibitors for Alzheimer’s disease. *Int. J. Alzheimers Dis*. 2012:728983. 10.1155/2012/728983 PMC3246720 22216416

[B12] Moir R. D. Lathe R. Tanzi R. E. (2018). The antimicrobial protection hypothesis of Alzheimer’s disease. *Alzheimer Dement.* 14 1602–1614. 10.1016/j.jalz.2018.06.3040 30314800

[B13] Nunez-Iglesias J. Liu C. C. Morgan T. E. Finch C. E. Zhou X. J. (2010). Joint genome-wide profiling of miRNA and mRNA expression in Alzheimer’s disease cortex reveals altered miRNA regulation. *PLoS One* 5:e8898. 10.1371/journal.pone.0008898 20126538 PMC2813862

[B14] Ono K. Hasegawa K. Naiki H. Yamada M. (2004). Curcumin has potent anti- amyloidogenic effects for Alzheimer’s beta-amyloid fibrils in vitro. *J. Neurosci. Res.* 75 742–750. 10.1002/jnr.20025 14994335

[B15] Pastore A. Raimondi F. Rajendran L. Temussi PA. (2020). Why does the Aβ peptide of Alzheimer share structural similarity with antimicrobial peptides? *Commun. Biol*. 3:135. 10.1038/s42003-020-0865-9 32193491 PMC7081199

[B16] Pereira J. B. Janelidze S. Ossenkoppele R. Kvartsberg H. Brinkmalm A. Mattsson-Carlgren N. (2021). Untangling the association of amyloid-β and tau with synaptic and axonal loss in Alzheimer’s disease. *Brain* 144 310–324. 10.1093/brain/awaa395 33279949 PMC8210638

[B17] Reddy P. H. Tonk S. Kumar S. Vijayan M. Kandimalla R. Kuruva C. S. (2017). A Critical evaluation of neuroprotective and neurodegenerative MicroRNAs in Alzheimer’s disease. *Biochem. Biophys. Res. Commun*. 483 1156–1165. 10.1016/j.bbrc.2016.08.067 27524239 PMC5343756

[B18] Siedlecki-Wullich D. Miñano-Molina A. J. Rodríguez-Álvarez J. (2021). microRNAs as early biomarkers of Alzheimer’s disease: A synaptic perspective. *Cells* 10:113. 10.3390/cells10010113 33435363 PMC7827653

[B19] Sokolik V. Berchenko O. Levicheva N. Shulga S. (2019). Anti-amyloidogenic Effect of MiR-101 in Experimental Alzheimer’s Disease. *Biotechnol. Acta* 12 41–49. 10.15407/biotech12.03.041

[B20] Sokolik V. Berchenko O. G. Shulga S. (2017). Comparative analysis of nasal therapy with soluble and liposomal forms of curcumin on rats with alzheimer’s disease model. *J. Alzheimer Dis. Parkinson.* 7:1000357. 10.4172/2161-0460.1000357

[B21] Sokolik V. V. Berchenko O. H. Kolyada O. K. Shulga S. M. (2021). Direct and indirect action of liposomal form of MIR-101 on cells in the experimental model of alzheimer’s disease. *Cytol. Genet.* 55 499–509. 10.3103/S0095452721060141

[B22] Sokolik V. V. Shulga S. M. (2015). Effect of curcumin liposomal form on angiotensin converting activity, cytokines, and cognitive characteristics of the rats with Alzheimer’s disease model. *Biotechnol. Acta* 8 48–55. 10.15407/biotech8.06.048

[B23] Sokolik V. V. Shulga S. M. (2016). Effect of curcumin on accumulation in mononuclear cells and secretion in incubation medium of Àβ40 and cytokines under local excess of Àβ42-homoaggregates. *Ukr. Biochem. J.* 88 83–91. 10.15407/ubj88.03.083 29235333

[B24] Vilardo E. Barbato C. Ciotti M. Cogoni C. Ruberti F. (2010). MicroRNA-101 regulates amyloid precursor protein expression in hippocampal neurons. *J. Biol. Chem.* 285 18344–18351. 10.1074/jbc.M110.112664 20395292 PMC2881760

